# Care management and leadership according to nurses’ perception in the hospital context of COVID-19

**DOI:** 10.1590/1980-220X-REEUSP-2024-0049en

**Published:** 2024-09-09

**Authors:** Pamela Gissi Lima, Geisa Colebrusco de Souza Gonçalves, Gabriela Marcellino de Melo Lanzoni, Alexandre Pazetto Balsanelli

**Affiliations:** 1 Universidade Federal de São Paulo, Escola Paulista de Enfermagem, São Paulo, SP, Brazil.; 2 Universidade Federal de Santa Catarina, Departamento de Enfermagem, Florianópolis, SC, Brazil.

**Keywords:** Leadership, Nursing, COVID-19, Nursing Care, Nursing Administration Research, Liderazgo, Enfermería, COVID-19, Atención de Enfermería, Investigación en Administración de Enfermería

## Abstract

**Objective:**

To understand how nursing care management occurred during the COVID-19 pandemic.

**Method:**

A qualitative study conducted at a university hospital in São Paulo, Brazil. The sample consisted of eight nurses who worked caring for patients who tested positive for COVID-19. Data collection was carried out through semi structured interviews about experiences in managing care in coping with the pandemic. Thematic analysis and interpretation based on psychodynamics of work were used in data analysis.

**Results:**

The results allowed constructing three thematic categories: The invisible that limits: biosafety, distress, uncertainty and fear of the pandemic, protecting oneself and ensuring the protection of others; Management work process instruments: team training, staff sizing, materials management, creative practice in the face of insufficiency; The competencies involved with the team, teamwork and leadership.

**Conclusion:**

Care management in COVID-19 was permeated by objective and subjective conditions, with situations of distress, pleasure, fear, insecurity and creative adaptation. Teamwork and leadership competencies, when present, can alleviate the distress that occurs in nursing work.

## RESUMEN

**Objetivo:**

Comprender cómo ocurrió la gestión del cuidado de enfermería en el contexto de la pandemia de COVID-19.

**Método:**

Estudio cualitativo, realizado en un hospital universitario de São Paulo, Brasil. La muestra estuvo conformada por ocho enfermeras que laboraron en la atención de pacientes positivos a COVID-19. La recolección de datos se realizó a través de entrevistas semiestructuradas sobre experiencias de gestión del cuidado frente a la pandemia. En el análisis de los datos se utilizó el análisis temático y la interpretación basada en la psicodinámica del trabajo.

**Resultados:**

Los resultados permitieron la construcción de tres categorías temáticas: Lo invisible que limita: bioseguridad, sufrimiento, incertidumbre y miedo a la pandemia, protegerse y velar por la protección de los demás; Instrumentos del proceso de trabajo gerencial: formación de equipos, dimensionamiento de personal, gestión de materiales, práctica creativa ante la insuficiencia; Las habilidades involucradas con el colectivo, el trabajo en equipo y el liderazgo.

**Conclusión:**

La gestión del cuidado durante la COVID-19 estuvo permeada por condiciones objetivas y subjetivas, con situaciones de sufrimiento, placer, miedo, inseguridad y adaptación creativa. El trabajo en equipo y las habilidades de liderazgo, cuando están presentes, pueden aliviar el sufrimiento en el trabajo de enfermería.

## INTRODUCTION

The onset of an outbreak of pneumonia of unknown cause in 2019 in the city of Wuhan, China, led to the discovery of a new coronavirus (SARS-CoV-2), identified as the etiological agent of severe acute respiratory syndrome, leading the World Health Organization (WHO) to declare the COVID-19 pandemic in March 2020^(1,2)^.

The health emergency and the increase in the number of cases have led to the need to manage resources and the health workforce in all healthcare services. Specifically in nursing, excessive workload management, the lack of personal protective equipment (PPE) to act in the face of the high transmissibility of the virus and continuing education of clinical management of patients have intensified the need for training and communication^(3)^.

Nursing represents the largest workforce in the health sector, accounting for 95% of the care that patients receive during their institutionalization^(4)^ and, therefore, performs essential work in the different healthcare services.

Nursing work can be understood based on five processes: care; management; educational; investigative; and political articulation^(5)^. This professional, as a formal leader of a team of nursing assistants and technicians, must be able to identify the presence or absence of characteristics that favor the professional practice of the different categories and implement actions that contribute to improving this environment and, consequently, patient care results^(4)^.

To manage care, nurses use technologies, knowledge, and various management tools to plan and organize individual and/or collective care, articulating management, care, teaching, research and political participation actions, work processes that often coexist in the same activity^(5,6)^.

Furthermore, considering psychodynamics of work, it is understood that, when working, i.e., when managing care, there is a subjective mobilization of workers, the experience in which individual and collective psychological resources are activated for a symbolic construction of meaning in the work context. Using these resources is intrinsically linked to the contribution and symbolic retribution dynamics, implying acknowledging workers. From these experiences, a person expands their capacity to feel, think and innovate to carry out work activities^(7,8)^.

Scientific research on healthcare workers’ mental health, linked to concerns about the managerial role, has been widely conducted. A mixed-methods study for resilience and well-being training for healthcare professionals working in high-pressure clinical environments aimed to expand these professionals’ capacity to manage difficult feelings caused by caring for illness, death and limited recovery, with the premise that, beyond the technical dimension, feelings of pleasure and satisfaction accompany healthcare work and should be recognized. The study concluded that program participants had a positive experience in well-being in the quality of relationships, communication and organizational culture, and emphasized the need for leadership commitment to provide time for participation in actions during the work period^(9)^. In a literature review on the managerial role of nursing during the COVID-19 pandemic, expansion and changes in the functions performed, critical decision-making and scarce resource management were identified, which caused anguish, concern about ensuring team well-being, equipment provision, support, communication, psychosocial service, training and learning, in addition to adequate staffing^(10)^.

Nationally, mental illness was identified during the COVID-19 pandemic in a survey carried out with healthcare professionals from different areas working on the front line, in which 61.6% of these workers were distress from mental distress and had a high rate of professional burnout, indicating negative repercussions of the pandemic and an imbalance between distress and pleasure^(11)^.

Although this research was conducted in Brazil, it considered workers at all levels of care. From this perspective, understanding how nurses managed care in the work process during a pandemic in hospital settings, how they provided and made health technologies available, considering each person’s and community’s needs at different moments in their lives, aiming at well-being, safety and autonomy, in addition to caring for the other workers who make up the team, is essential, since there is little evidence of this nature, therefore requiring understanding not only from an objective point of view, but from the symbolic construction of meanings, the subjectivities intrinsic to the work activity^(7,8)^.

The COVID-19 pandemic has triggered problems in healthcare systems ranging from financing and infrastructure to care management. In the managerial role of nursing, the focus has been on its technical dimensions. A study conducted in India demonstrated that horizontal management was successful, especially in revealing the fragility of existing healthcare infrastructure^(12)^. In Iran, they developed a management model called “nursing in coronavirus crisis model”. Through a Scientific Nursing Committee, they determined the responsibilities of producing educational materials, in addition to managerial and supervisory actions^(13)^.

It is known that nursing care management is permeated by work processes and mainly involves management and direct care actions^(5)^, but that, beyond the objective point of view, it is permeated by subjectivities. A scarce literature on nurses’ experiences related to care management during the COVID-19pandemic shows that, in addition to being a little explored topic, it encourages developing research that can consolidate the lessons learned from this important global health crisis regarding care management, especially from the perspective of the larger health workforce, nursing and beyond prescriptive work. In this context, the object of research was management of nurses’ care in the context of COVID-19, with the guiding question: how did nursing care management occur during the COVID-19 pandemic in a Brazilian university hospital from nurses’ perspective? The objective was to understand how nursing care management occurred during the COVID-19 pandemic from nurses’ perspective.

## METHOD

### Study Design

This is a qualitative study that followed the COnsolidated criteria for REporting Qualitative research (COREQ)^(14)^ guidelines in its conduction and dissemination. It is part of a multicenter research project, carried out at ten university hospitals in Brazil, called “*Avaliação do Cuidado de Enfermagem a Pacientes COVID-19 em Hospitais Universitários Brasileiros*”, funded by MCTIC/CNPq/FNDCT/MS/SCTIE/Decit Call 07/2020, Process 402392/2020-5.

### Local

The university hospital in this study is located in the state of São Paulo, Brazil, and offers multidisciplinary inpatient and emergency care. It is a large, highly complex tertiary hospital that mainly serves patients from the Brazilian Healthcare system (SUS – *Sistema Único de Saúde*).

### Population and Selection Criteria

The population consisted of nurses who worked in providing care to patients who tested positive for COVID-19 during the pandemic in different hospital clinical units.

### Sample Definition

The sample consisted of eight nurses who worked in different hospital units and who were a nurse, had experience in care management, assistance and/or management in units during the COVID-19 pandemic for at least three months and who received a patient who tested positive for COVID-19, in addition to having participated in the quantitative stage of the multicenter project mentioned previously.

### Data Collect

During the multicenter project, nurses who agreed to participate in the research were subsequently consulted to consent to a semi-structured interview. Those who responded positively were personally invited to participate using the contact details previously provided by a nurse (email or messaging app). In total, 30 nurses were invited. Additionally, 23 nurses were contacted by email and seven by messaging app and voice call, with only nine receiving feedback and scheduling appointments, one of which was excluded after data collection. The interview was conducted via Google Meet^®^, respecting health guidelines regarding social distancing and data collection in a virtual environment^(15)^.

The interview script included nine questions about nursing care management in dealing with the COVID-19 pandemic, and participants were invited to report on their experience, routine of care work (direct care provision) for people with COVID-19, care organization, technologies, instruments and tools used. Critical-reflective questions were also asked about strategies that could be used to improve care organization by nurses and their teams to strengthen nursing leadership, ensure patient and team safety, and quality of care. Furthermore, characterization information was collected from participants regarding sex, age, level of education, work unit, the length of time working as a nurse in the institution, in the unit and with patients with COVID-19, the main characteristic of the activity performed (care and/or management), workload and whether they had other institutional relationships.

The interviews took place between September 2021 and January 2022, and lasted an average of 24 minutes (minimum of 17 minutes and 45 seconds; maximum of 32 minutes and 28 seconds). Data collection was carried out by the main researcher of this study, who received training and was based on a manual developed by the multicenter research team for qualitative research. The interviewer and participants did not know each other previously. The interviews were recorded only in audio and completely transcribed, and the audios were transformed into raw texts for material analysis.

### Data Analysis and Treatment

For data analysis, the described procedures of thematic analysis were used^(16)^. First, the interviews were organized into raw texts in Microsoft Word^®^. The material from each interview and from the set of interviews was read carefully and in an inquisitive manner. Subsequently, a directive reading was carried out to find units of meaning and to extract excerpts from the interviews, whereas maintaining the meaning of the speech. The units of meaning guided the construction of the empirical categories, through charts from each interview that, separated by similarity of ideas, composed the preliminary categories. These charts were regrouped in the set of interviews, in which similarities, divergences or contradictions could be analyzed. Subsequently, second-order interpretation was carried out. The empirical categories were contemplated in light of the theoretical framework, work, work process in health and nursing and psychodynamics of work, approaching Christophe Dejours’s theoretical and conceptual constructs about pleasure and distress, prescribed and real work, considering the subjectivities that permeate the objective work reported by nurses^(7,8)^. The construction of the final categories was carried out through a meeting for reaching consensus among the authors. The results are presented in thematic categories regarding nursing care management during the COVID-19 pandemic.

### Ethical Aspects

The study followed the ethical precepts for research with human beings, in accordance with Resolution 466 of 2012 of the Brazilian National Council for Research Ethics (CONEP – *Conselho Nacional de Ética em Pesquisa*), in addition to the guidelines of Letter 1 of 2021 of CONEP^(15)^ for research involving data collection in a virtual environment. It was approved by the teaching and research host institution Research Ethics Committee and by the co-participating institution, field of study, under opinions and years of approval, respectively: 4,381,848, 2020 and 5,452,034, 2022, 2022. Participants consented by signing the Informed Consent Form, made available prior to data collection. To maintain confidentiality and anonymity, nurses’ responses were identified according to the order of the interview, using the letter “I”, followed by the numbers from 1 to 9 to identify the speech of the same interviewee.

## RESULTS

All interviewees were female nurses, aged between 23 and 44 years, with an average age of 36 years. Regarding training, four were experts (n = 4; 50%); two held a master’s degrees (n = 2; 25%); one held a doctoral degree (n = 1; 12.5%); and one was specialization student (n = 1; 12.5%). The length of time working as a nurse ranged from two to 21 years, with an average of 11 years. The length of time working at the hospital, where the study was conducted, ranged from one and a half years to 21 years. Two nurses had another employment relationship in addition to the study site (n = 2; 25%).

As for the units, four worked in obstetrics hospitalization (n = 4; 50%), one, in Intensive Care Unit (n = 1; 12.5%), and one, in each of the following sectors: clinical ward (n = 1; 12.5%); hemodynamics (n = 1; 12.5%); and nephrology and dialysis (n = 1; 12.5%). The length of time working in these current units varied between less than one year and ten years. The weekly workload was 24 to 36 hours, and one (n = 1; 12.5%) works extra shifts in the same hospital in addition to this workload.

Concerning the main characteristic of work activities, six nurses reported care activities (n = 6; 75%), and two, care and management activities (n = 2; 25%). The length of time spent working in care management for patients affected by SARS-CoV-2 ranged from less than one year to one year and six months, i.e., since the beginning of the pandemic and the arrival of COVID-19 positive patients at the hospital, considering data collection from September 2021.

In the interview material analysis, three categories were constructed, “The invisible that limits: biosafety, distress, uncertainty and fear of the pandemic, protecting oneself and ensuring the protection of others”, “Management work process instruments: team training, staff sizing, materials management, creative practice in the face of insufficiency”, “The competencies involved with the team, teamwork and leadership”, which are presented below.

*The invisible that limits: biosafety, distress, uncertainty and fear of the pandemic, protecting oneself and ensuring the protection of others*

In their speeches, nurses mentioned distress during the COVID-19 pandemic, uncertainty and fear of contracting the virus, and doubts about the technical dimension, due to the lack of studies on care management for patients with COVID-19. They also pointed out aspects related to team mental health, with the perception of increased stress and anxiety and the negative influence on work practices. Biosafety was widely discussed by interviewees, whether due to the need for new PPE, requisition, insufficiency, or a feeling of insecurity regarding the quality of this equipment. The lack of PPE could culminate in exposure of healthcare professionals and other hospitalized patients, generating insecurity. The perception of insecurity changed throughout the COVID-19 pandemic. Initially, there was a shortage of PPE, which was later remedied, but insecurity about working conditions still remained, as highlighted in the explanatory excerpts:

*It was very exhausting and besides all the stress of an unknown disease. We also didn’t know much about how to manage a patient.(I4)*

*So, in the beginning, besides being challenging, there was a lot of uncertainty as to whether we were really providing the best care.(I5)*

*At the beginning, which was in March 2020, I was very scared because everything was new, so, in the first few days, there were cases in other places, but not in the hospital itself, the feeling I had was that at any moment we could come into contact with the invisible.(I7)*

*Sometimes, we receive material that apparently has gone through an approval process by HICC [Hospital Infection Control Commission], and we see that it is low-quality material that exposes professionals to greater risks. So, we end up constantly negotiating, talking to the HICC, talking to the board of directors to be able to collect it. We notify the materials and equipment.(I3)*

*The team has to try to be aware at all times of the need to wear the correct donning.(I5)*

*We couldn’t ignore the mask every time and there was a time when there was a shortage of material and we couldn’t get an apron.(I4)*

*I believe that, since the end of last year, stress has increased a lot, and nurses are the population that has had the highest increase in anxiety levels, so it is stressful.(I9)*

*Management work process instruments: team training, staff sizing, materials management, creative practice in the face of insufficiency*

In the context of care management, the technical dimension of the management process stands out from excerpts, adapted in a creative manner. There was distress and concern about not always having what was needed, such as people and materials, but there was pleasure in recognizing and doing what was necessary, even if in an adapted and creative manner. In people management, it was necessary to resize and train the team, experienced people and new hires, actions that are fundamental to guaranteeing care in a dynamic and unprecedented scenario. In relation to materials management, which were not always available in sufficient quantity and quality, it was necessary to create and adapt several devices, such as material conference forms, cushions made with orthopedic cotton and sheets, “electronic baby monitors” to monitor patients in isolation, breastfeeding pillows to place pregnant women with COVID-19 in prone position, in addition to using a messaging application (WhatsApp^®^) to speed up communication. Below are some excerpts that highlight these findings:

*The flow was changed several times, so there was a need for training. We have to reinforce this with the teams all the time.(I3)*

*At first, it was very difficult, because we had an ICU [Intensive Care Unit] that was much smaller and we were expecting an increase in flow, so we had to train a lot of “raw” people, with no experience in the area, and we who had more experience in the ICU were responsible for training.(I4)*

*Also, the issue of staffing, because it took us by surprise, so in terms of coordination, management, we had to adapt within that scenario.(I5)*

*You create a lot of things because you don’t have the things. The truth is, you create things because you don’t have the right ones. So, you create different types of bandages, you create ways to make bandages, you create new uses for things. I remember once I made a baby bottle with a tube and a half-full water bottle. One thing we do a lot is make our own shower water bottle, cutting a collection bottle to make an urinal, because there’s no urinal in the unit either, and the bottles are already intended for making showers, so there’s no bottle... we end up adapting a lot.(I1)*

*I think there needs to be an improvement in the issue of these resources, to be able to guarantee higher quality care, with less stress for us too, because, when we don’t have the appropriate material, the appropriate medication, our care is also compromised and it’s something that we are very concerned about.(I3)*

*The competencies involved with the team, teamwork and leadership*

When managing care during the COVID-19 pandemic, nurses emphasized two necessary and essential competencies during this period: teamwork and leadership. Teamwork was cited as important, considered a present and essential competency for nursing, but which still requires improvement to organize care. Regarding nurses’ leadership competency, participants indicated a concept of a guiding leader, active in the team, who makes decisions and does so based on scientific knowledge. They also indicated recognizing aspects that can strengthen and expand both nurse leadership and teamwork during the COVID-19 pandemic, such as encouraging collaboration, caring for workers’ mental health, professional appreciation, scientific knowledge, and institutional support, aspects that can mitigate the perception of distress at work, as per excerpts:

*Teamwork(lack), because many times a technician wants to take care of their patient, they don’t want to help their colleague, and sometimes you ask them and they get angry, but they will also need help from their colleague because they will forget things when they enter the room. So, I think teamwork is one of the things that needs to be improved.(I1)*

*Working together, sharing not only among the nursing team, I think we need to identify the moment when other team members need to take action.(I5)*

*Leadership is improved when you discuss with the team.(I8)*

*People think that being a leader is just about delegating, but it’s about working together, being close to the team, trying to understand, having a vision of the whole, being able to work with the particularities, it’s... making decisions all the time. We have to manage resources, equipment, materials, people and have good communication.(I3)*

*Being present (a leader), who is by the team’s side, especially in the most difficult moments, which I think brings important security to the team, who knows how to listen, right? That just because they are there in a leadership position doesn’t mean they have to be authoritarian.(I5)*

*The nurse, as a leader, is the one who creates an entire environment for the team, an entire emotional environment for the team.(I6)*

*The issue of leadership depends a lot on that; your nurses need to have maturity and also self-confidence, self-knowledge, basing all these transformations of recognition of the profession.(I9)*

## DISCUSSION

Nursing care management is an essential part of their work in hospital services. During the COVID-19 pandemic, the results of this research highlighted a dynamic conflict between the organization of one’s own work and psychological functioning, work, mental health, pleasure and distress that goes beyond the technical dimension of the processes in which it is inserted. When exploring aspects that direct and indirect care organization for patients during the COVID-19 pandemic, nurses related: distress during the practice of care; uncertainty and fear of the pandemic; responsibility and insecurity in protecting oneself and ensuring the protection of others; doubts about the unusual technical dimension of work in COVID-19; perception that nursing workers’ mental health has worsened; the need to review staffing levels; increased frequency of training; and in materials management, given their insufficiency, creativity and adaptation to cope with patient care. From this perspective, two competencies stood out: teamwork and leadership, which are involved with the team and, when present, contribute to alleviating the distress encountered at work.

Uncertainty and fear of the pandemic stood out in survey results, ranging from fear of contracting the virus to doubts about the best practices for the moment, generating stress and anguish that, although they are valid feelings and sensations, could not keep them away from work. During the pandemic, the intensification of work, exhausting and non-stop workdays, inadequate conditions, and insufficient quality and quantity of protective materials continued. In a survey conducted in the Brazilian context, it was identified that the pandemic intensified existing structural problems, especially in the Brazilian healthcare system, affecting the health workforce, with repercussions in socioeconomic inequalities reproduced in the workplace, precariousness, lack of protection in execution of care, fragility in biosafety, culminating in the high rate of illness and deaths among health workers^(17)^.

The results of this research are similar to those identified in a cross-sectional observational study, in which indicators of pleasure and distress of health workers during the COVID-19 pandemic were identified. Regarding distress at work, the study identified that professional burnout and lack of recognition were classified as severe and critical, respectively, and, in relation to pleasure, professional fulfillment and freedom of expression were classified as satisfactory and critical, respectively, with emphasis on highly demanding work and low social support of nursing technicians and assistants^(11)^.

It is not possible to manage care without objective dimensions, such as sufficient numbers and qualification of people as well as material resources. The feeling of overload, insufficient training for care, and lack of material resources negatively impact both patients and professionals, since patients may not receive adequate care, and professionals may not be assured of the value and safety of their practice. Difficulties also identified in another study during the COVID-19 pandemic, such as managing excessive workload, lack of PPE, and the constant need for continuing education^(3)^, are similar to the findings of this study.

Conflict between distress and pleasure was identified, in the possibility of providing care, even if in an adapted and creative manner. Instruments were created and adapted to improve care, use equipment for another purpose, make more use of available technology, reinvigorating the value and, therefore, the pleasure at work. Technology can facilitate training professionals, carried out remotely, access to electronic medical records and even patient monitoring after hospital discharge, through teleconsultations^(18)^, demonstrating that technology during the COVID-19 pandemic was an ally in managing care for in-hospital patients.

In the case of Brazilian nursing, work’s objective difficulties are compounded by the lack of material resources and PPE and the struggle for a minimum wage, which has been ongoing for decades and has been intensified during the COVID-19 pandemic. Extrapolated to the national level, it refers to the search for decent pay for nursing work, undertaken by class organizations that are trying to achieve structural contours of the relationship between nursing work and capital^(19)^. The perception of devaluation permeates the context of the work of nurses who verbalized the need for greater “professional appreciation”, whether based on insufficient remuneration, the number of professionals and/or lack of materials to carry out the necessary care.

It is worth noting that, in the work relationships established between workers and the organization, a return is expected that goes beyond wage and recognition of the quality of what is done, the social importance to which it is linked, as well as feeling safe to perform activities, subjective expectations that workers carry. Pleasure and distress counterbalance each other, and when there is recognition materialized by decent working conditions and safety, they can be positive, but when they are not met, difficulties of daily life can be characterized as distress^(8)^. In the interviews, objective and subjective working conditions were cited as insufficient by nurses. Professional recognition was also considered critical in another study^(11)^, and in this study, although limited to nurses’ work, lack of recognition and worsening of nursing workers’ mental health were identified.

The nursing team’s mental health was addressed by nurses, especially in the increased perception of anxiety and stress. An integrative review found that healthcare professionals have high levels of stress and tension at work, as they work on the front line of care, and highlighted the importance of carrying out mental health interventions with these professionals^(20)^. Another study also identified, during the COVID-19 pandemic, wear, physical exhaustion and stress, in addition to physical and psychological pressure, which contributed to feelings of insecurity and fear of the pandemic^(21)^.

From this perspective, it is no coincidence that nursing has presented and perceived high rates of illness at work, as mentioned by the interviewees in this study, supporting other studies that have focused on workers’ mental distress. Work and health workers are characterized and challenged by being an emotional job, often doubly challenging, by hiding the feelings generated by the practice of care and managing them, in order to comply with organizational standards^(8)^ and with the social pressures of being the “heroes” of the pandemic.

Moreover, psychodynamics of work highlights that “if the task carries a symbolic content, if the work allows, despite the limitations of reality and organization, an inventive exercise of the bodies, it becomes a source of pleasure and sublimation”^(22:91)^. Nurses’ subjective mobilization in care management during the COVID-19 pandemic was permeated by distress, uncertainty and fear, but it also offered an opportunity to reinvent themselves, change flows and adopt measures that allowed the creation, even in the most protocolary work, such as health work, the use of equipment for purposes other than those for which they were idealized, reinforcing the idea that, in instituted and instituting care management, distress and pleasure counterbalance each other.

Furthermore, biosafety was a concern cited by nurses, linked to the highly transmissible characteristics of the COVID-19 virus, the need to adopt new PPE, which is not always available in adequate quantity and quality, new ways of organizing flows, entry and exit from the room and unit, greater control of the risk of biological exposure, with the need to raise awareness among all professionals and to coordinate with other hospital sectors, highlighting the need for communication within the team and the organization. It is noteworthy that communication was cited in research as the primary competency in combating the pandemic and managing the crisis, in managing people and in training for adopting constant changes^(3)^. A Chinese hospital has adopted the PDCA (Plan, Do, Check, Act) quality tool to manage care during the COVID-19 pandemic, facilitating the definition of “clean and contaminated areas”, with greater staff awareness of them^(23)^. PDCA proved to be a simple, practical and low-cost solution for professionals’ safety, especially in donning and doffing, concerns cited by interviewees, highlighting the need to build collective defensive systems to deal with situations of distress in nursing work.

It is worth remembering that, even in the most prescriptive and regulated work, such as healthcare, workers mobilize individual and collective defense strategies. In the case of the pandemic, understanding, based on prior knowledge, ways to deal with insecurity and lack of materials in a creative manner must occur based on organizational agreements produced, individually and collectively^(8)^, in patient care management, considering professionals’ needs.

Furthermore, the aspect that deserves to be highlighted and that influenced nursing work’s objective and subjective conditions was workload, which increased, according to nurses’ perception, during the COVID-19 pandemic, worsened by the hiring of recent graduates at the hospital and the lack of scientific evidence to address the specificities of COVID-19 illness. Actions to resize and reallocate the team were necessary, since patients who tested positive required specific care and spent more time on the process of donning and doffing. They cited team training as essential to standardize practices and offer greater safety in patient care and in the execution of work by professionals. Many changes were perceived in care management by interviewees, whose majority of activities were care or management. It is worth noting that nurses reported majority activities, but in work processes that coexisted with care, management, teaching, research, and political participation activities^(5)^.

The pandemic scenario further increased the perception that, in these work processes, teamwork and leadership competencies were necessary, competencies related to collective work that, when present, help to alleviate distress in the workplace. Teamwork was cited as essential in facing the COVID-19 pandemic and that nurses need to be aligned with the nursing team to ensure safe and quality care, in addition to being able to lead so that communication occurs effectively and generates a relationship of respect and trust among all. This result is in line with a study in Europe that brought together the experience of nurses who worked on the front line, and they cited the importance of teamwork, not only in nursing, but also from an interprofessional perspective^(24)^.

Nurses recognized that, although present, these competencies needed improvement. It is worth highlighting that nursing’s professional competencies go beyond their technical performance and professionals’ ability to promote, in addition to care, social changes^(25)^. In a summary of evidence from a systematic review of the experiences of nursing managers in dealing with the COVID-19 pandemic, five themes emerged from studies as possibilities for dealing with the challenges of this period: the expanding and constantly changing management function, the need to ensure team well-being, communication, support received and development, and learning^(10)^. These challenges can be linked to a lack of teamwork and leadership, in line with the findings of this research.

Leadership competency was cited as enabling care management with the necessary quality and safety, and is similar to what was presented in the literature review on offering support, developing learning and ensuring team well-being^(10)^. The greater the scientific knowledge of nurses in their professional performance, the greater their empowerment in decision-making and, consequently, the greater the trust of other professionals who share hospital care. Communication also permeates this competency, since nurses are alongside the team and must actively participate in the processes, and it is up to them to include and value team contributions. The literature also highlights, in addition to what was brought by nurses, that leadership can strengthen professional appreciation, minimum and adequate working conditions and recognition by nursing autonomy institutional management^(25)^.

By sharing the subjectivities involved in work activities during the COVID-19 pandemic, nurses recognize themselves and other nursing professionals as team members, establishing mutual relationships of trust and solidarity^(8)^. The opposite, lack of recognition, can lead to illness, frustration and distress. From this perspective, leadership competencies can enable, in addition to physical safety, the psychological safety involved in work.

In a survey conducted, invisibility and insensitivity of leadership, perceived by nurses, in relation to the exposure of themselves or family members in meeting patient care demands during the COVID-19 pandemic, generated a feeling of being dispensable to organization, reinforcing that the different areas of an organization must be aligned with the values of care not only for patients, but for the health team^(26)^. Thus, the understanding is reinforced that, when work activity is understood only from the perspective of its bodily functioning, workers are considered instrument that drives the organizational driving force^(8)^.

As predicted by theoretical constructions of psychodynamics of work, between the prescribed and the effective^(7,8)^, nurses reported, in their interviews, a web of activities experienced in care management during the COVID-19 pandemic that generated fear and anguish about contamination of themselves and others, creative processes in the face of the scarcity of material resources, raising the need for competencies involved with the team, especially by highlighting teamwork and leadership, reinforcing the understanding that work is, at the same time, an objective action permeated by subjectivities.

A limitation of this study is data collection, since most of the nurses who participated in the multicenter project and were contacted did not respond to the invitation or refused to participate. This refusal may be related to the overload of their own work or lack of interest in making their contributions to the research, aspects that were not analyzed. Another point is the remote mediation of the interview, because, although it is justifiable due to the data collection period and ethically supported, it may generate reports with less detail and participants may feel strange in their responses mediated by technology. Regarding the findings, due to the qualitative nature of this research, it is emphasized that these are understandings based on personal and subjective reports about the phenomenon, not reaching data saturation. Furthermore, qualitative data interpretation is subject to the plurality of analytical understanding, although the strategy of consensus meetings was used for categorization.

The findings of this research, in the context of the greatest health crisis in history this century, the COVID-19 pandemic, identified multiple aspects that influenced effective and prescriptive action in care management in hospital settings from nurses’ perspective. It is recommended that healthcare services ensure, in emergency situations, safe work environments, in addition to adequate remuneration, staff sizing and material resources for patient care, horizontal communication, demonstrating management’s commitment and support, with an action plan and goals recognized by the team. In this analysis, it is highlighted that health work is conceived in the connection between activity and subjectivity; the activity is not only technical, because, despite being concrete, is irreducible and needs to be explored for its subjectivity.

## CONCLUSION

It is concluded that many aspects, both objective and subjective, influenced the care management carried out by nurses. Work was configured between the mediation of the prescribed and the effective, in addition to the technical dimension and biosafety, permeated by subjectivity, uncertainty and fear of the pandemic, in an attempt to balance distress and pleasure.

Furthermore, management work instruments, staff sizing, team training and sufficient material resources, considered fundamental for safe and quality care, although based on technical rules, are permeated by subjectivity, as the necessary resources for care are not guaranteed, resulting in distress and, at the same time, mobilization of creativity.

In nursing care management, collective work competencies are required, such as teamwork and leadership. Leadership and teamwork go hand in hand; these competencies can equip workers, contributing to reducing distress at work. Leadership must be close, offer support, communication, contribute to learning and ensure team well-being.
