# Supplementary material for: Care management and leadership according to nurses’ perception in the hospital context of COVID-19
Source: Rev Esc Enferm USP. 2024 Sep 9;58:e20240049. doi: 10.1590/1980-220X-REEUSP-2024-0049en (PMC11391903; doi:10.1590/1980-220X-REEUSP-2024-0049en)
Supplement: Translation

# Translation: Gestão do cuidado e liderança na percepção de enfermeiras no contexto hospitalar da COVID-19

**DOI:** 10.1590/1980-220X-REEUSP-2024-0049pt

**Authors:** Pamela Gissi Lima, Geisa Colebrusco de Souza Gonçalves, Gabriela Marcellino de Melo Lanzoni, Alexandre Pazetto Balsanelli

**Affiliations:** 1 Universidade Federal de São Paulo, Escola Paulista de Enfermagem, São Paulo, SP, Brasil.; 2 Universidade Federal de Santa Catarina, Departamento de Enfermagem, Florianópolis, SC, Brasil.

**Keywords:** Liderança, Enfermagem, COVID-19, Cuidados de Enfermagem, Pesquisa em Administração de Enfermagem

## Abstract

**Objetivo:**

Compreender como ocorreu a gestão do cuidado de enfermagem no contexto da pandemia de COVID-19.

**Método:**

Estudo qualitativo, realizado em hospital universitário em São Paulo, Brasil. A amostra constituiu-se de oito enfermeiras que atuaram no cuidado de pacientes positivados para COVID-19. A coleta de dados foi realizada por meio de entrevista semiestruturada sobre experiências na gestão do cuidado no enfrentamento da pandemia. Na análise dos dados, utilizaram-se a análise temática e a interpretação a partir da psicodinâmica do trabalho.

**Resultados:**

Os resultados permitiram a construção de três categorias temáticas: O invisível que limita: biossegurança, sofrimento, incerteza e medo da pandemia, proteger-se e garantir a proteção do outro; Instrumentos do processo de trabalho gerencial: treinamento da equipe, dimensionamento do pessoal, gestão de materiais, a prática criativa diante da insuficiência; as competências implicadas com o coletivo, trabalho em equipe e liderança.

**Conclusão:**

A gestão do cuidado na COVID-19 foi permeada por condições objetivas e subjetivas, com situações de sofrimento, prazer, medo, insegurança e adaptação criativa. As competências de trabalho em equipe e liderança, quando presentes, podem amenizar o sofrimento vigente no trabalho da enfermagem.

## INTRODUÇÃO

O início de um surto de pneumonia de causa desconhecida em 2019 na cidade de Wuhan, na China, levou à descoberta de um novo coronavírus (SARS-CoV-2), identificado como agente etiológico da Síndrome Respiratória Aguda Grave, levando a Organização Mundial da Saúde (OMS) decretar a pandemia de COVID-19 em março de 2020^(1,2)^.

A emergência sanitária e o aumento do número de casos desdobraram-se em necessidades de gerir recursos e a força de trabalho em saúde em todos os serviços de saúde. Especificamente na enfermagem, a gestão de carga de trabalho excessiva, a falta de equipamentos de proteção individual (EPIs) para atuação diante da alta transmissibilidade do vírus e a educação continuada de condução clínica dos pacientes intensificaram a necessidade de treinamento e de comunicação^(3)^.

A enfermagem corresponde à maior força de trabalho na área da saúde, sendo responsável por 95% da assistência que os pacientes recebem durante sua institucionalização^(4)^ e, portanto, executa trabalho essencial nos diferentes serviços de saúde.

O trabalho da enfermagem pode ser compreendido com base em cinco processos: assistencial; gerencial; educativo; investigativo; e de articulação política^(5)^. Esse profissional, enquanto líder formal de uma equipe de auxiliares e técnicos de enfermagem, deve ser capaz de identificar a presença ou a ausência de características que favorecem a prática profissional das diferentes categorias e implementar ações que contribuam para a melhoria desse ambiente e, consequentemente, dos resultados do cuidado ao paciente^(4)^.

Para gerir o cuidado, o enfermeiro utiliza tecnologias, conhecimentos, além de diversas ferramentas de gestão para o planejamentos e a organização dos cuidados, individuais e/ou coletivos, na articulação de ações gerenciais, assistenciais, de ensino, pesquisa e de participação política, processos de trabalho que muitas vezes coexistem na mesma atuação^(5,6)^.

Ainda, considerando a psicodinâmica do trabalho, compreende-se que, ao trabalhar, ou seja, ao gerir o cuidado, há a mobilização subjetiva do trabalhador, a experiência na qual os recursos psicológicos individuais e coletivos são acionados para uma construção simbólica de significado no contexto laboral. A utilização desses recursos está intrinsecamente ligada à dinâmica de contribuição e retribuição simbólica, implicando o reconhecimento do trabalhador. A partir dessas experiências, a pessoa amplia sua capacidade de sentir, pensar e inovar para a realização das atividades laborais^(7,8)^.

Pesquisas científicas sobre a saúde mental dos trabalhadores de saúde, atreladas à preocupação do papel gerencial, têm sido amplamente conduzidas. Estudo de métodos mistos para treinamento de resiliência e bem-estar para profissionais de saúde atuantes em ambientes clínicos de alta pressão objetivou expandir a capacidade desses profissionais gerenciarem sentimentos difíceis provocados pelo cuidado de doenças, morte e recuperação limitada, com a premissa de que, para além da dimensão técnica, sentimentos de prazer e satisfação acompanham o trabalho em saúde e devem ser reconhecidos. O estudo concluiu que os participantes do programa tiveram uma experiência positiva no bem-estar na qualidade dos relacionamentos, na comunicação e na cultura organizacional, e enfatizaram a necessidade de compromisso da liderança para oferecer tempo para participação de ações durante o período do trabalho^(9)^. Em revisão de literatura acerca do papel gerencial da enfermagem durante a pandemia de COVID-19, identificaram-se expansão e mudanças nas funções desempenhadas, tomadas de decisões críticas e gestão de recursos escassos, que provocaram angústia, preocupação em garantir bem-estar da equipe, oferta de equipamentos, apoio, comunicação, serviço psicossocial, treinamento e aprendizagem, além de dimensionamento adequado de pessoal^(10)^.

No cenário nacional, identificou-se, durante a pandemia de COVID-19, o adoecimento mental em pesquisa realizada com profissionais de saúde de diferentes áreas atuantes na linha de frente, em que 61,6% desses trabalhadores estavam em sofrimento mental e apresentavam alta taxa de esgotamento profissional, indicando repercussões negativas da pandemia e desequilíbrio entre sofrimento e prazer^(11)^.

Apesar da pesquisa citada ter sido conduzida no território brasileiro, considerou trabalhadores de todos os níveis de atenção. Nessa perspectiva, compreender como se deu a gestão do cuidado pelos enfermeiros no processo de trabalho durante uma pandemia no contexto hospitalar, como realizaram o provimento e a disponibilização das tecnologias de saúde, considerando as necessidades de cada pessoa e coletividade nos diferentes momentos da sua vida, visando ao bem-estar, à segurança e à autonomia, além do cuidado com os demais trabalhadores que compõe a equipe, é fundamental, uma vez que existem poucas evidências dessa natureza, demandando, portanto, compreender não apenas do ponto de vista objetivo, mas da construção simbólica de significados, das subjetividades intrínsecas à atividade de trabalho^(7,8)^.

A pandemia de COVID-19 deflagrou problemas nos sistemas de saúde relacionados desde o financiamento e infraestrutura até a gestão do cuidado. No papel gerencial da enfermagem, o foco tem sido nas suas dimensões técnicas. Em pesquisa realizada na Índia, demonstrou que a gestão horizontal foi exitosa, especialmente ao revelar a fragilidade da infraestrutura de saúde existente^(12)^. No Irã, desenvolveram um modelo de gestão denominado “enfermagem no modelo de crise do coronavírus”. Por meio de um Comitê Científico de Enfermagem, determinaram as responsabilidades de produzirem materiais educativos, além das ações gerenciais e de supervisão^(13)^.

Sabe-se que a gestão do cuidado de enfermagem é permeada pelos processos de trabalho, e envolve majoritariamente ações de gerenciamento e assistência direta^(5)^, mas que, para além do ponto de vista objetivo, é permeada pelas subjetividades. A escassez de literatura sobre as experiências dos enfermeiros relacionadas à gestão do cuidado durante a pandemia de COVID-19 mostra que, além de ser tema pouco explorado, suscita o desenvolvimento de pesquisas que possam consolidar as lições aprendidas desta importante crise sanitária global no que concerne à gestão do cuidado, sobretudo na perspectiva da maior força de trabalho em saúde, à enfermagem e para além do trabalho prescritivo. Nesse contexto, o objeto da pesquisa foi a gestão do cuidado dos enfermeiros no contexto da COVID-19, tendo como pergunta norteadora: como ocorreu a gestão do cuidado de enfermagem no contexto da pandemia de COVID-19em um hospital universitário brasileiro na visão dos próprios enfermeiros? O objetivo foi compreender como ocorreu a gestão do cuidado de enfermagem no contexto da pandemia de COVID-19 na perspectiva dos enfermeiros.

## MÉTODO

### Desenho do Estudo

Trata-se de estudo de abordagem qualitativa, que seguiu as diretrizes dos *COnsolidated criteria for REporting Qualitative research* (COREQ)^(14)^ na condução e divulgação. É parte integrante de pesquisa multicêntrica, realizada em dez hospitais universitários do Brasil, denominada “Avaliação do Cuidado de Enfermagem a Pacientes COVID-19 em Hospitais Universitários Brasileiros”, financiada pela Chamada MCTIC/CNPq/FNDCT/MS/SCTIE/Decit Nº 07/2020, Processo nº 402392/2020-5.

### Local

O hospital universitário deste estudo está localizado no estado de São Paulo, Brasil, e oferece atendimento multiprofissional à saúde de internação e urgência/emergência. Trata-se de hospital terciário, de grande porte e alta complexidade que atende majoritariamente pacientes do Sistema Único de Saúde (SUS).

### População e Critérios de Seleção

A população foi constituída por enfermeiros que atuavam na assistência aos pacientes positivados para COVID-19 durante a pandemia em unidades clínicas diversas do hospital.

### Definição da Amostra

A amostra foi constituída por oito enfermeiras que trabalhavam em unidades diversas do hospital e que obedeceram aos seguintes critérios de inclusão: ser enfermeiro, ter experiência em gestão do cuidado, assistência e/ou gerência nas unidades durante a pandemia de COVID-19 por, no mínimo, três meses e que recebeu paciente positivado para COVID-19, além de ter participado da etapa quantitativa do projeto multicêntrico citado previamente.

### Coleta de Dados

Durante a realização do projeto multicêntrico, os enfermeiros que aceitaram participar da pesquisa foram consultados, *a posteriori,* para consentir com uma entrevista semiestruturada. Aos que sinalizaram positivamente, foi realizado convite pessoal em contato previamente disponibilizado por ele (e-mail ou aplicativo de mensagem). No total, foram convidados 30 enfermeiros. Além disso, 23 enfermeiros foram contatados por e-mail e, sete, por aplicativo de mensagem e ligação de voz, obtendo retorno e agendamento de apenas nove, sendo que uma foi excluída após a coleta de dados. A entrevista foi realizada via plataforma *Google Meet* ^®^, respeitando as orientações sanitárias de distanciamento e de coleta de dados em ambiente virtual^(15)^.

O roteiro de entrevista comtemplava nove perguntas acerca da gestão do cuidado do enfermeiro no enfrentamento da pandemia de COVID-19, sendo convidado a relatar sobre a sua experiência, rotina de trabalho assistencial (prestação de cuidados diretos) às pessoas com COVID-19, organização do cuidado, tecnologias, instrumentos e ferramentas utilizados. Também foram realizadas perguntas crítico-reflexivas sobre as estratégias que poderiam ser utilizadas para melhorar a organização do cuidado pelo enfermeiro e sua equipe, para fortalecer a liderança do enfermeiro, garantir a segurança do paciente e da equipe, além da qualidade na assistência. Ainda, informações de caracterização foram coletadas dos participantes sobre sexo, idade, nível de formação, unidade de atuação, tempo de trabalho como enfermeiro na instituição, na unidade e com pacientes com COVID-19, característica majoritária da atividade desenvolvida (assistencial e/ou gerencial), carga horária e se possuía outros vínculos institucionais.

As entrevistas ocorreram entre os meses setembro de 2021 e janeiro de 2022, e tiveram duração média de 24 minutos (mínima de 17 minutos e 45 segundos; máxima de 32 minutos e 28 segundos). A coleta de dados foi realizada pela pesquisadora principal deste estudo, a qual recebeu capacitação e teve como base um manual desenvolvido pela equipe da pesquisa multicêntrica para pesquisas qualitativas. Entrevistadora e participantes não se conheciam previamente. As entrevistas foram gravadas somente em áudio e completamente transcritas, e os áudios foram transformados em textos brutos para a análise do material.

### Análise e Tratamento dos Dados

Para a análise dos dados, foram utilizados os procedimentos descritos de análise temática^(16)^. Primeiramente, as entrevistas foram organizadas em textos brutos no *Microsoft Word* ^®^. Realizou-se leitura atenta e indagativa do material de cada entrevista e do conjunto das entrevistas. Posteriormente, procedeu-se à leitura diretiva, para encontrar unidades de significado e, desprender excertos das entrevistas, com manutenção do sentido da fala. As unidades de significado guiaram a construção das categorias empíricas, por meio de quadros de cada entrevista que, separados por similaridade de ideias, compuseram as categorias preliminares. Esses quadros foram reagrupados no conjunto das entrevistas, em que similaridades, divergências ou contradições puderam ser analisadas. Na sequência, foi realizada a interpretação de segunda ordem. As categorias empíricas foram contempladas à luz do referencial teórico, trabalho, processo de trabalho em saúde e enfermagem e psicodinâmica do trabalho, com aproximações dos construtos teóricos e conceituais de Christophe Dejours acerca do prazer e sofrimento, do trabalho prescrito e real, considerando as subjetividades que permeiam o trabalho objetivo relatado pelas enfermeiras^(7,8)^. A construção das categorias finais passou por reunião de consenso entre os autores. Os resultados estão apresentados em categorias temáticas acerca da gestão do cuidado de enfermagem durante a pandemia de COVID-19.

### Aspectos Éticos

O estudo seguiu os preceitos éticos para pesquisa com seres humanos, em conformidade com a Resolução nº 466 de 2012 do Conselho Nacional de Ética em Pesquisa (CONEP), além das orientações da Carta nº 1 de 2021 do CONEP^(15)^ para pesquisas que envolvem coleta de dados em ambiente virtual. Foi aprovado pelo Comitê de Ética em Pesquisa da instituição sede de ensino e pesquisa e pela instituição coparticipante, campo do estudo, sob os números dos pareceres e anos de aprovação, respectivamente: 4.381.848, 2020 e 5.452.034, 2022. Houve o consentimento dos participantes mediante assinatura do Termo de Consentimento Livre e Esclarecido, disponibilizado previamente à coleta dos dados. Para manutenção do sigilo e anonimato, as respostas das enfermeiras foram identificadas de acordo com a ordem da entrevista, utilizando a letra “E”, seguida dos números de 1 a 9 para identificar a fala da mesma entrevistada.

## RESULTADOS

Todas as entrevistadas eram enfermeiras, mulheres, com idade entre 23 e 44 anos e média de 36 anos. Em relação à formação, quatro eram especialistas (n = 4; 50%); duas eram mestres (n = 2; 25%); uma era doutora (n = 1; 12,5%); e uma estava cursando especialização (n = 1; 12,5%). O tempo de atuação como enfermeira variou de dois a 21 anos, com média de 11 anos. O tempo de trabalho no hospital, local do estudo, variou de um ano e meio a 21 anos. Duas enfermeiras possuíam outro vínculo de trabalho além do local do estudo (n = 2; 25%).

Quanto às unidades, quatro atuavam na internação de obstetrícia (n = 4; 50%), uma, em Unidade de Terapia Intensiva (n = 1; 12,5%), uma, em cada um dos setores: enfermaria clínica (n = 1; 12,5%); hemodinâmica (n = 1; 12,5%); e nefrologia e diálise (n = 1; 12,5%). O tempo de trabalho nessas unidades atuais variou entre menos de um ano e dez anos. A carga horária semanal era de 24 a 36 horas, e uma (n = 1; 12,5%) realiza plantões extras no mesmo hospital além dessa carga horária.

Com relação à característica principal das atividades de trabalho, seis enfermeiras relataram atividades assistenciais (n = 6; 75%), e duas, assistenciais e gerenciais (n = 2; 25%). O tempo de trabalho na gestão do cuidado dos pacientes acometidos pelo SARS-CoV-2 variou entre menos de um ano e um ano e seis meses, ou seja, desde o início da pandemia e da chegada de pacientes positivos por COVID-19 no hospital, considerando a coleta de dados a partir de setembro de 2021.

Na análise do material das entrevistas, foram construídas três categorias, “O invisível que limita: biossegurança, sofrimento, incerteza e medo da pandemia, proteger-se e garantir a proteção do outro”, “Instrumentos do processo de trabalho gerencial: treinamento da equipe, dimensionamento do pessoal, gestão de materiais, a prática criativa diante da insuficiência”, “As competências implicadas com o coletivo, trabalho em equipe e liderança”, que estão apresentadas abaixo.

*O invisível que limita: biossegurança, sofrimento, incerteza e medo da pandemia, proteger-se e garantir a proteção do outro*

As enfermeiras referiram, em seus discursos, sofrimento durante a pandemia de COVID-19, incerteza e medo de contrair o vírus, dúvidas na dimensão técnica, devido à escassez de estudos de gestão do cuidado de pacientes com COVID-19. Ainda, apontaram aspectos relacionados à saúde mental da equipe, com a percepção de aumento de estresse e ansiedade e da influência negativa na prática laboral. A biossegurança foi amplamente abordada pelas entrevistadas, seja pela necessidade de novos EPIs, requisição, insuficiência e sentimento de insegurança da qualidade desses equipamentos. A falta de EPI poderia culminar na exposição dos profissionais de saúde e demais pacientes internados, gerando insegurança. A percepção de insegurança mudou ao longo da pandemia de COVID-19. No início, havia escassez de EPIs, que foi posteriormente sanada, ainda permanecendo a insegurança sobre as condições de trabalho, conforme se destaca nos excertos elucidativos:

*Foi muito desgastante e fora todo o estresse de uma doença desconhecida. A gente também não sabia muito manejar o paciente.(E4)*

*Então, o começo, além de ser desafiador, tinha bastante insegurança, se a gente estava realmente prestando a melhor assistência.(E5)*

*No começo, que foi em março de 2020, eu fiquei com muito medo porque era tudo novo, então, nos primeiros dias que tinham casos em outros locais, mas no hospital mesmo não tinha, a sensação que tinha era que a todo momento a gente podia entrar em contato com o invisível.(E7)*

*Às vezes, vem um material que aparentemente passa por um processo de aprovação da CCIH [Comissão de Controle de Infecção Hospitalar], que a gente vê que é algum material de baixa qualidade, que expõe os profissionais a riscos maiores, então a gente acaba o tempo todoarticulando, falando com a CCIH, falando com a diretoria pra poder fazer esse recolhimento. A gente faz a notificação dos materiais, equipamentos.(E3)*

*A equipe tem que tentar se conscientizar o tempo inteiro, da necessidade de uma paramentação correta.(E5)*

*A gente não podia desprezar a máscara toda vez e teve uma época que estava faltando material e a gente não podia pegar avental.(E4)*

*Eu acredito que, do final do ano passado pra cá, o estresse aumentou muito, e o enfermeiro é a população que mais aumentou o nível de ansiedade, então é estressante.(E9)*

*Instrumentos do processo de trabalho gerencial: treinamento da equipe, dimensionamento do pessoal, gestão de materiais, a prática criativa diante da insuficiência*

No contexto da gestão do cuidado, destaca-se dos excertos a dimensão técnica do processo gerencial, adaptado de forma criativa. Houve sofrimento e preocupação por nem sempre ter o que era preciso, como pessoas e materiais, mas prazer em reconhecer e fazer o que era necessário, mesmo que de forma adaptada e criativa. Na gestão de pessoas, foi preciso redimensionar, treinar a equipe, pessoas com experiência e recém-contratados, ações fundamentais para garantir o cuidado em cenário dinâmico e sem precedentes. Em relação à gestão de materiais, nem sempre disponíveis em quantidade e qualidade suficientes, houve necessidade de criação e adaptação de diversos dispositivos, como impressos de conferência material, coxins produzidos com algodão ortopédico e lençol, “babá eletrônica” para monitorar pacientes em isolamento, almofada de amamentação para colocar gestantes com COVID-19 em posição prona, além de utilizarem aplicativo de mensagens (*WhatsApp* ^®^) para agilidade da comunicação. Abaixo alguns excertos evidenciam esses achados:

*O fluxo foi mudado várias vezes, então teve a necessidade de treinamento. O tempo todo a gente tem que ficar reforçando com as equipes.(E3)*

*No começo, foi muito difícil, porque a gente tinha uma UTI [Unidade de Tratamento Intensivo] que era bem menor e a gente estava esperando um aumento do fluxo, então a gente teve que treinar muita gente “crua”, assim, sem experiência nenhuma na área, e a gente que tinha mais experiência na UTI que ficava responsável pelos treinamentos.(E4)*

*E a questão também de dimensionamento de pessoal, porque pegou a gente meio que de surpresa, então a parte da coordenação, da gerência, a gente teve que se adaptar dentro daquele cenário.(E5)*

*Você cria várias coisas, porque você não tem as coisas. A verdade é essa, você cria as coisas porque você não tem as certas. Então, você cria tipos de curativos diferentes, você cria formas de fazer o curativo, você cria novos usos para as coisas. Eu lembro uma vez que eu fiz uma mamadeira com uma sonda e uma garrafa d’água pela metade assim. Uma coisa que a gente faz muito é fazer a própria garrafa d’água de chuveirinho, cortar um frasco coletor para fazer papagaio, porque também não tem papagaio na unidade, e as garrafas já são destinadas para fazer o chuveirinho do banho, então falta garrafa… a gente acaba adaptando bastante.(E1)*

*Eu acho que precisaria ter uma melhoria na questão desses recursos, para poder garantir o cuidado de maior qualidade, com menos estresse pra gente também, porque, quando a gente não tem o material adequado, o medicamento adequado, a nossa assistência também fica prejudicada e é uma coisa que a gente fica muito preocupada.(E3)*

*As competências implicadas com o coletivo, trabalho em equipe e liderança*

Na gestão do cuidado durante a pandemia de COVID-19, as enfermeiras enfatizaram duas competências necessárias, imprescindíveis neste período: trabalho em equipe e liderança. O trabalho em equipe foi citado como algo importante, considerado competência presente e primordial para a enfermagem, mas que ainda requer aprimoramento para organizar o cuidado. Com relação à competência de liderança do enfermeiro, as participantes indicaram uma concepção de líder guia, atuante na equipe, que toma decisões e a faz com base em conhecimento científico. Ainda, indicaram reconhecer aspectos que podem fortalecer e ampliar tanto a liderança do enfermeiro quanto o trabalho em equipe no contexto da pandemia de COVID-19, como estímulo à colaboração, cuidado à saúde mental dos trabalhadores, valorização profissional, conhecimento científico e apoio institucional, aspectos que podem mitigar a percepção de sofrimento no trabalho, conforme excertos:

*Trabalho em equipe(falta), porque muitas vezes o técnico quer cuidar do paciente dele, não quer ajudar o colega, e às vezes você pede e ele fica com raiva, mas ele também vai precisar de ajuda do colega porque ele vai esquecer as coisas quando entrar no quarto. Então, acho que trabalho em equipe é uma das coisas que precisa ser melhorada.(E1)*

*Trabalho em conjunto, do compartilhar não só entre equipe de enfermagem, eu acho que de a gente identificar o momento que é preciso que outros membros da equipe entrem em ação.(E5)*

*A liderança é melhorada quando você discute com a equipe.(E8)*

*As pessoas acham que ser líder é a questão de só delegar, mas é o trabalhar junto, estar próximo à equipe, tentar entender, ter uma visão do todo, né, conseguir trabalhar as particularidades, é… tomada de decisão o tempo todo. A gente tem que gerenciar recursos, gerenciar equipamentos, materiais, pessoas e ter boa comunicação.(E3)*

*Estar presente(líder), que está ao lado da equipe, principalmente nos momentos de maior dificuldade, que acho que isso traz uma segurança importante pra equipe, que sabe ouvir, né? Que não é porque ele tá ali em uma posição de liderança que tem que ser algo autoritário.(E5)*

*O enfermeiro, como líder, ele que cria todo um ambiente pra equipe, todo um emocional pra equipe.(E6)*

*A questão da liderança vai muito daquilo; seus enfermeiros precisam ter amadurecimento e também autoconfiança, autoconhecimento, baseando todas essas transformações de reconhecimento da profissão.(E9)*

## DISCUSSÃO

A gestão do cuidado pela enfermagem é parte essencial do seu trabalho nos serviços hospitalares. No contexto da pandemia de COVID-19, os resultados desta pesquisa evidenciaram uma dinâmica do conflito entre organização do próprio trabalho e funcionamento psíquico, trabalho, saúde mental, prazer e sofrimento que extrapola a dimensão tecnicista dos processos aos quais se insere. Ao explorar os aspectos que influenciaram a organização do cuidado direto e indireto aos pacientes durante a pandemia de COVID-19, as enfermeiras relacionaram: sofrimento durante a prática de cuidado; incerteza e medo da pandemia; responsabilidade e insegurança de se proteger e garantir a proteção do outro; dúvida na dimensão técnica inabitual do trabalho na COVID-19; percepção de que a saúde mental dos trabalhadores de enfermagem piorou; a necessidade de rever o dimensionamento do pessoal; maior frequência de treinamentos; e na gestão de materiais, diante de sua insuficiência, a criatividade e a adaptação para dar conta do cuidado dos pacientes. Nessa perspectiva, houve destaque para duas competências, trabalho em equipe e liderança, implicadas com o coletivo que, quando presentes, contribuem para amenizar o sofrimento encontrado no trabalho.

A incerteza e o medo da pandemia se sobressaíram nos resultados da pesquisa, variando desde o medo de contrair o vírus até dúvidas acerca das melhores práticas para o momento, gerando estresse e angústia que, embora sejam sentimentos e sensações válidas, não poderiam afastá-los do trabalho. Durante a pandemia, perpetuaram-se a intensificação do trabalho, jornadas desgastantes e sem pausas, condições inadequadas, materiais de qualidade e quantidade insuficiente para proteção. Em pesquisa realizada no contexto brasileiro, identificou-se que a pandemia intensificou problemas estruturais já existentes, sobretudo no sistema de saúde brasileiro, afetando a força de trabalho em saúde, com reflexo nas desigualdades socioeconômicas reproduzidas no trabalho, precarização, desproteção na execução do cuidado, fragilidade em biossegurança, culminando no elevado adoecimento e mortes dos trabalhadores de saúde^(17)^.

Os resultados desta pesquisa se assemelham ao identificado em pesquisa observacional transversal, em que puderam identificar indicadores de prazer e sofrimento dos trabalhadores de saúde durante a pandemia de COVID-19. Quanto ao sofrimento no trabalho, o estudo identificou que esgotamento profissional e falta de reconhecimento foram classificados como grave e crítico, respectivamente, e, em relação ao prazer, a realização profissional e a liberdade de expressão foram classificadas como satisfatória e crítica, respectivamente, com destaque para o trabalho de alta exigência e de baixo apoio social dos técnicos e auxiliares de enfermagem^(11)^.

Não é possível realizar a gestão do cuidado sem que haja dimensões objetivas para tal, como pessoas em quantidade e qualificação suficiente, além de recursos materiais. A sensação de sobrecarga, a formação insuficiente para o atendimento e a falta de recursos materiais impactam negativamente tanto o paciente quanto os profissionais, uma vez que o paciente pode não receber assistência adequada, e o profissional, não ter assegurados o valor e a segurança para sua prática. Dificuldades também identificadas em outra pesquisa durante a pandemia de COVID-19, como gerir a carga de trabalho excessiva, a falta de EPIs e a constante necessidade de educação continuada^(3)^, aproximam-se dos achados deste estudo.

Identificou-se o conflito entre sofrimento e o prazer, na possibilidade de cuidar mesmo que de forma adaptada e criativa. Instrumentos foram criados e adaptados para melhorar o cuidado, utilizar um equipamento com outra finalidade, usufruir mais da tecnologia disponível, revigorando o valor e, portanto, o prazer no trabalho. A tecnologia pode facilitar o treinamento dos profissionais, realizado remotamente, o acesso ao prontuário eletrônico e até mesmo o monitoramento do paciente após a alta hospitalar, por meio de teleconsultas^(18)^, demonstrando que a tecnologia durante a pandemia de COVID-19 foi uma aliada na gestão do cuidado dos pacientes em cuidado hospitalar.

No caso da enfermagem brasileira, somam-se concomitantemente às dificuldades objetivas do trabalho a falta de recursos materiais e de EPIs e a luta pelo piso salarial, que perdura há décadas, e foi intensificada durante a pandemia de COVID-19. Extrapolada ao nível nacional, refere-se à busca pelo pagamento digno do trabalho da enfermagem, empreendida por organizações de classe que tentam alcançar contornos estruturais da relação trabalho da enfermagem e capital^(19)^. A percepção de desvalorização permeia o contexto do trabalho das enfermeiras que verbalizaram a necessidade de maior “valorização profissional”, seja com base na remuneração insuficiente, na quantidade de profissionais e ou na falta de materiais para realizar o cuidado necessário.

Cabe destacar que, nas relações de trabalho estabelecidas entre trabalhadores e organização, esperam-se uma contrapartida que extrapola o salário e o reconhecimento da qualidade do que é realizado, da importância social a qual se vincula, bem como de sentir-se seguro para executar as atividades, expectativas subjetivas que o trabalhador carrega. Prazer e sofrimento se contrabalançam, e quando há reconhecimento materializado por condições de trabalho e segurança dignas, podem ser positivas, mas quando não atendidas, as dificuldades do dia a dia podem caracterizarem-se como sofrimento^(8)^. Nas entrevistas, condições objetivas e subjetivas de trabalho foram citadas como insuficientes pelas enfermeiras. O reconhecimento profissional também foi considerado crítico em outro estudo^(11)^, e neste estudo, embora circunscrito ao trabalho das enfermeiras, reconheceram-se falta de reconhecimento e piora da saúde mental dos trabalhadores de enfermagem.

A saúde mental da equipe de enfermagem foi abordada pelas enfermeiras, sobretudo no aumento da percepção de ansiedade e estresse. Revisão integrativa constatou que profissionais de saúde possuem altos níveis de estresse e tensão no trabalho, ao aturem na linha de frente do cuidado, e destacou a importância de realizar intervenções em saúde mental com esses profissionais^(20)^. Outro estudo também identificou, durante a pandemia de COVID-19, exaustão, esgotamento físico e estresse, além da pressão física e psicológica, que corroboraram para os sentimentos de insegurança e medo da pandemia^(21)^.

Nessa perspectiva, não é mero acaso que a enfermagem tenha apresentado e percebido altos índices de adoecimento no trabalho, conforme citado pelas entrevistadas deste estudo, corroborando as demais pesquisas que tiveram como objeto o sofrimento mental dos trabalhadores. Ao trabalho e aos trabalhadores de saúde, recaem a característica e o desafio de ser um trabalho emocional, muitas vezes os desafiando duplamente, ao ocultar os sentimentos gerados pela prática do cuidado e gerenciá-los, de modo a cumprir com as normas organizacionais^(8)^ e com as pressões sociais de serem os “heróis” da pandemia.

Ainda, a psicodinâmica do trabalho destaca que “se a tarefa carrega um conteúdo simbólico, se o trabalho permite, apesar das limitações do real e da organização, um exercício inventivo dos corpos, ele se torna fonte de prazer e sublimação”^(22:91).^ A mobilização subjetiva das enfermeiras na gestão do cuidado durante a pandemia de COVID-19 foi permeada pelo sofrimento, pela incerteza e pelo medo, mas também ofereceu uma oportunidade de se reinventarem, mudarem fluxos e adotarem medidas que permitiram a criação, mesmo no trabalho mais protocolar, como o da saúde, a utilização de equipamentos para outras finalidades distintas das que foram idealizadas, reforçando a ideia de que, na gestão do cuidado instituído e instituinte, sofrimento e prazer se contrabalançam.

Ainda, a biossegurança foi uma preocupação citada pelas enfermeiras atrelada às características de alta transmissibilidade do vírus da COVID-19, necessidade de adoção de novos EPIs, nem sempre disponíveis em quantidade e qualidade adequadas, novas formas de organização dos fluxos, entrada e saída do quarto e da unidade, maior controle do risco de exposição biológica, com necessidade de conscientização de todos os profissionais e da articulação com os demais setores do hospital, evidenciando a necessidade de comunicação na equipe e na organização. Destaca-se que a comunicação foi citada em pesquisa como a competência primordial no combate à pandemia e gestão da crise, na gestão das pessoas e no treinamento de adoção das mudanças constantes^(3)^. Hospital chinês adotou a ferramenta da qualidade PDCA (*Plan, Do, Check, Act*) para gerir o cuidado no contexto da pandemia de COVID-19, facilitando a definição de “áreas limpas e contaminadas”, com maior conscientização da equipe sobre elas^(23)^. A PDCA mostrou ser uma solução simples, prática e de baixo custo para a segurança dos profissionais, em especial na paramentação e desparamentação, preocupações citadas pelas entrevistadas, evidenciando a necessidade de construção de sistemas defensivos coletivos para lidar com as situações do sofrimento no trabalho da enfermagem.

Cabe lembrar que, mesmo no trabalho mais prescritivo e regrado, como o da saúde, os trabalhadores mobilizam estratégias de defesa individuais e coletivas. No caso da pandemia, compreender, a partir do conhecimentos prévios, formas de lidar com a insegurança e com a falta de material de maneira criativa deve ocorrer a partir de acordos organizativos produzidos, individuais e coletivamente^(8)^, na gestão do cuidado dos pacientes, considerando as necessidades dos profissionais.

Ainda, o aspecto que merece destaque e que influenciou as condições objetivas e subjetivas do trabalho da enfermagem foi a carga de trabalho, que aumentou, na percepção das enfermeiras, durante a pandemia de COVID-19, agravando-se pela contratação de recém-formados no hospital e pela escassez de evidências científicas para atender às especificidades do adoecimento por COVID-19. Ações para redimensionamento e realocação da equipe foram necessárias, visto que os pacientes positivados necessitavam de cuidado específico, dispêndio de maior tempo no processo de paramentação e desparamentação. Citaram o treinamento da equipe como essencial para padronizar as práticas e oferecer maior segurança no cuidado dos pacientes e na execução do trabalho pelos profissionais. Muitas mudanças foram percebidas na gestão do cuidado das entrevistadas, cujas atividades majoritárias eram assistenciais ou gerenciais. Cabe elucidar que as enfermeiras relataram atividades majoritárias, mas em processos de trabalho coexistentes de atividades assistenciais, gerenciais, de ensino, pesquisa e de participação política^(5)^.

O cenário da pandemia aumentou ainda mais a percepção de que, nesses processos de trabalho, foram necessárias competências de trabalho em equipe e de liderança, competências relacionadas ao trabalho coletivo que, quando presentes, ajudam a aliviar o sofrimento no ambiente de trabalho. O trabalho em equipe foi citado como essencial no enfrentamento da pandemia de COVID-19 e que enfermeiros precisam estar alinhados à equipe de enfermagem para garantir um cuidado seguro e de qualidade, além de serem capazes de liderar de modo que a comunicação ocorra de maneira eficaz e gere uma relação de respeito e confiança entre todos. Esse resultado é consoante a estudo na Europa que trouxe a experiência de enfermeiros que atuaram na linha de frente, e os mesmos citaram a importância do trabalho em equipe, não só da enfermagem, mas também na perspectiva interprofissional^(24)^.

As enfermeiras reconheceram que, embora presentes, essas competências precisavam de aprimoramento. Cabe destacar que as competências profissionais da enfermagem extrapolam a sua atuação técnica e a capacidade do profissional promover, além da assistência, mudanças sociais^(25)^. Em síntese de evidências de revisão sistemática acerca das experiências dos gerentes de enfermagem no enfrentamento da pandemia de COVID-19, cinco temas emergiram dos estudos como possibilidades de lidar com os desafios desse período: a função gerencial em expansão e constante mudança, a necessidade de garantir o bem-estar da equipe, a comunicação, o apoio recebido e desenvolvimento, e aprendizado^(10)^. Esse desafios podem ser atrelados à falta de trabalho em equipe e de liderança, alinhando-se aos achados desta pesquisa.

A competência de liderança foi citada como viabilizadora da gestão do cuidado com qualidade e segurança necessária, e se assemelha ao trazido na revisão de literatura sobre oferecer apoio, desenvolver aprendizagem e garantir o bem-estar da equipe^(10)^. Quanto maior o conhecimento científico do enfermeiro na sua atuação profissional, maior empoderamento na tomada de decisão e, consequentemente, maior confiança dos demais profissionais que compartilham o cuidado no hospital. A comunicação também permeia essa competência, uma vez que o enfermeiro está ao lado da equipe e deve participar ativamente nos processos, cabendo a ele incluir e valorizar a contribuição do coletivo. A literatura destaca, ainda, para além do que foi trazido pelas enfermeiras, que a liderança pode fortalecer a valorização profissional, as condições mínimas e adequadas de trabalho e o reconhecimento pela gestão institucional da autonomia da enfermagem^(25)^.

Ao compartilharem as subjetividades implicadas nas atividades de trabalho durante a pandemia de COVID-19, as enfermeiras reconhecem em si e nos outros profissionais de enfermagem como membros de uma coletividade, estabelecendo relações mútuas de confiança e de solidariedade^(8)^. O oposto, a falta de reconhecimento, pode gerar adoecimento, frustração e sofrimento. Nessa perspectiva, a competência de liderança pode viabilizar, para além da segurança física, a segurança psíquica implicada no trabalho.

Em pesquisa realizada, invisibilidade e insensibilidade da liderança, percebidas pelos enfermeiros, em relação à exposição de si mesmo ou de familiares no atendimento das demandas de cuidado ao paciente durante a pandemia de COVID-19, geraram sentimento de serem dispensáveis para a organização, reforçando que as diferentes áreas de uma organização devem estar alinhadas aos valores de cuidado não só para o paciente, mas para a equipe de saúde^(26)^. Assim, reforça-se a compreensão de que, quando a atividade de trabalho é compreendida apenas a partir de seu funcionamento corporal, o trabalhador é considerado um instrumento que impulsiona a força motriz organizacional^(8)^.

Conforme previsto pelas construções teóricas da psicodinâmica do trabalho, entre o prescrito e o efetivo^(7,8)^, as enfermeiras relataram, em suas entrevistas, uma trama de atividades vividas na gestão do cuidado em saúde durante a pandemia de COVID-19 que gerou medo e angústias acerca da contaminação de si e de outros, processos criativos diante da escassez de recursos materiais, suscitando a necessidade de competências implicadas com o coletivo, sobretudo ao destacar o trabalho em equipe e a liderança, reforçando a compreensão de que o trabalho é, ao mesmo tempo, ação objetiva permeada de subjetividades.

Destaca-se como limitação deste estudo a coleta de dados, visto que a maioria dos enfermeiros participantes do projeto multicêntrico contactados não responderam ao convite ou se recusaram a participar, recusa que pode estar relacionada ao momento de sobrecarga do próprio trabalho ou desinteresse em tecer suas contribuições na pesquisa, aspectos não analisados. Outro apontamento é a mediação remota da entrevista, pois, embora seja justificável pelo período da coleta de dados e respaldada eticamente, pode gerar relatos com menor riqueza de detalhes e estranhamento das participantes em suas respostas mediadas pela tecnologia. Em relação aos achados, devido ao carácter qualitativo da pesquisa, reforça-se que são compreensões a partir de relatos pessoais e subjetivos acerca do fenômeno, não alcançando a saturação dos dados. Ainda, a interpretação dos dados qualitativos está sujeita à pluralidade de compreensão analítica, embora tenha sido utilizada a estratégia de reuniões de consenso para a categorização.

Os achados desta pesquisa no contexto da maior crise sanitária da história neste século, a pandemia de COVID-19, identificou múltiplos aspectos que influenciaram a ação efetiva e prescritiva na gestão do cuidado no ambiente hospitalar na perspectiva das enfermeiras. Recomenda-se que os serviços de saúde garantam, em situações emergenciais, ambientes de trabalho seguros, além de remuneração adequada, dimensionamento e recursos materiais para o cuidado do paciente, comunicação horizontal, demonstrando o compromisso e o apoio da gestão, com plano de ação e metas reconhecidas pelo coletivo. Nesta análise, destaca-se que o trabalho em saúde é concebido na conexão entre atividade e subjetividade; a atividade não é apenas técnica, pois, apesar de concreta, é irredutível e precisa ser explorada pela sua subjetividade.

## CONCLUSÃO

Conclui-se que muitos aspectos, objetivos e subjetivos, influenciaram a gestão do cuidado realizada pelas enfermeiras. O trabalho se configurou entre a mediação do prescrito e do efetivo, além da dimensão técnica e a biossegurança, permeada pela subjetividade, incerteza e medo da pandemia, na tentativa de equilibrar sofrimento e prazer.

Ainda, instrumentos do trabalho gerencial, dimensionamento, treinamento da equipe e recursos materiais suficientes, considerados fundamentais para uma assistência segura e de qualidade, embora com base em regras técnicas, são permeados pela subjetividade, ao não ter garantido os recursos necessários para o cuidado, havendo sofrimento e, ao mesmo tempo, a mobilização da criatividade.

Na gestão do cuidado de enfermagem, são requeridas competências implicadas com o coletivo, como trabalho em equipe e liderança. A liderança e o trabalho em equipe andam juntos; essas competências podem instrumentalizar os trabalhadores, contribuindo na redução dos sofrimentos presentes no trabalho. A liderança deve estar próxima, oferecer apoio, comunicação, contribuir com a aprendizagem e garantir o bem-estar da equipe.
